# Validation and confirmation of the brazilian version of the PinQ questionnaire in children with lower urinary tract dysfunction

**DOI:** 10.1590/1984-0462/2025/43/2024322

**Published:** 2025-12-12

**Authors:** Angélica Quintino, Ricardo Marcondes de Mattos, Jorge Pompemaier, Marcela Leal da Cruz, Antonio Macedo

**Affiliations:** a Universidade Federal de São Paulo, São Paulo, SP, Brazil.; b Centro de Apoio à Criança com Anomalia Urológica, Núcleo de Urologia Pediátrica, São Paulo, SP, Brazil.

**Keywords:** Dysfunction, Lower urinary tract symptoms, Urinary incontinence, Quality of life, Disfunção, Sintomas do trato urinário inferior, Incontinência urinária, Qualidade de vida

## Abstract

**Objective:**

The objective of this study was to validate the Continence Specific Pediatric Quality Of Life Measurement Tool (PinQ) questionnaire into Portuguese and compare its effectiveness with the Dysfunctional Voiding Symptom Score (DVSS) questionnaire in assessing the quality of life in children with lower urinary tract dysfunction (LUTD).

**Methods:**

The PinQ questionnaire was translated into Portuguese using Beaton's methodology, which consists of six phases: (1) Initial Translation; (2) Synthesis; (3) Back Translation; (4) Final Version; (5) Prefinal Version Testing; and (6) Presentation. Each patient received 15 sessions, consisting of one evaluation and application of questionnaires and 14 treatment sessions (sacral neurostimulation + behavioral therapy + central stabilization, diaphragm and transverse abdominal muscle recruitment and pelvic floor muscle activation), with the questionnaire reapplied at the end. The scores obtained from both questionnaires before and after treatment were used to compare treatment efficacy and validate the PinQ questionnaire.

**Results:**

A total of 300 sessions were conducted with 20 patients, each receiving 15 weekly sessions, with an average age of 8.95 years. The analysis of the PinQ questionnaire showed an improvement of symptoms (Score before: mean 37.7; deviation±15.9 and Score after: mean 26.8; deviation±12.9 with p=0.0051), as well as the DVVSS questionnaire (Score before: mean 11, deviation 3.4 and Score after: mean 7.7, deviation 4 with p=0.0055), with a significant improvement in the quality of life of children as assessed by both questionnaires.

**Conclusions:**

This study confirmed the effectiveness of a Portuguese-language tool that evaluates the real impact of quality of life in children and adolescents with LUTD.

## RESUMO

**Objetivo:**

Validar o questionário Continence Specific Pediatric Quality Of Life Measurement Tool (PinQ) para o português e comparar sua eficácia com o questionário Dysfunctional Voiding Symptom Score (DVSS) na avaliação da qualidade de vida em crianças com disfunção do trato urinário inferior.

**Métodos:**

O questionário PinQ foi traduzido para o português utilizando a metodologia de Beaton, que consiste em seis fases: 1) tradução inicial; 2) síntese; 3) retrotradução; 4) versão final; 5) teste da versão pré-final; 6) apresentação. Cada paciente recebeu 15 sessões, consistindo em uma avaliação e aplicação de questionários e 14 sessões de tratamento (neuroestimulação sacral + terapia comportamental + estabilização central, recrutamento do diafragma e dos músculos abdominais transversos e ativação dos músculos do assoalho pélvico), com o questionário reaplicado ao final. As pontuações obtidas em ambos os questionários antes e depois do tratamento foram utilizadas para comparar a eficácia do tratamento e validar o questionário PinQ.

**Resultados:**

Foram realizadas 300 sessões com 20 pacientes com idade média de 8,95 anos, cada um recebendo 15 sessões semanais. A análise do questionário PinQ mostrou melhora dos sintomas (escore antes: média 37,7; desvio±15,9; e escore depois: média 26,8, desvio±12,9 com p=0,0051), assim como a do questionário DVVSS (escore antes: média 11, desvio 3,4; e escore depois: média 7,7, desvio 4 com p=0,0055), com melhoria significativa na qualidade de vida das crianças avaliada por ambos os questionários.

**Conclusões:**

Este estudo confirmou a eficácia de um instrumento em língua portuguesa que avalia o real impacto da qualidade de vida em crianças e adolescentes com disfunção do trato urinário inferior.

## INTRODUCTION

The International Children's Continence Society (ICCS)^1^ considers lower urinary tract dysfunction (LUTD) to be the presence of urinary symptoms and disorders during the bladder filling and/or emptying phases, not due to neurological alterations or bladder and urethra malformations.^2^ LUTD is estimated to affect about 10% of children and adolescents worldwide, ranking second among the most common urinary disorders in childhood, affecting approximately 6.4–10.3% of children by age 8.^1,3^ In Brazil, among children aged 3–9 years, the prevalence is approximately 24.2%, with 11.2% in boys and 35.8% in girls. Non-neurogenic urinary disorders in childhood predominate in children aged 7 years (3–22%) and 10 years (0.4–8.4%). LUTD is strongly related to psychosocial problems, behavioral disorders, attention and learning issues, and social exclusion, affecting the quality of life of both children and their families.^4-8^

Recognizing the need for a more specific instrument to assess and measure the impact of LUTD on the quality of life of affected children, Bower et al.^9^ and Bower^10^ developed and tested the "Continence Specific Pediatric Quality of Life Measurement Tool (PinQ)." In Brazil, the quality of life of children with LUTD is still evaluated using generic questionnaires that fail to identify the real impact of urinary symptoms on psychosocial relationships and self-esteem. The most widely used questionnaire is the Dysfunction Voiding Symptoms Score (DVSS), originally developed and validated by Farhat et al.^11^ in Canada and later translated into Portuguese and validated by Calado et al.^12^

It is a method for assessing urinary and fecal habits in children, with recommended cutoff points of six for girls and nine for boys. The questionnaire was administered on the first day of treatment and repeated at the end of treatment on the 15th day. A cutoff point ≥ 48 was considered to indicate good quality of life, and a cutoff point below 48 was considered to indicate impaired quality of life for the population studied.^13^

In Brazil, the quality of life of children with LUTD is still assessed using generic questionnaires, which fail to identify the true impact of urinary symptoms on psychosocial relationships and self-esteem. Therefore, a preliminary version of this questionnaire was developed, administered not only to children but also to parents, to assess its reliability.^14^

This study aims to validate the PinQ questionnaire for Portuguese and, as a secondary objective, to compare its effectiveness with the DVSS questionnaire in assessing quality of life in children with LUTD.

## METHOD

This is a cross-sectional study conducted at the specialized pediatric urology outpatient clinic of the Center for Support of Children with Urological Anomalies (CACAU) at the Federal University of São Paulo, approved by Ethics Committee CEP/UNIFESP no.: 1211/2021; CAAE: 52689921.4.0000.5505, involving literate children aged 6–18 years with LUTD and who agreed to sign the informed consent form, from May 2021 to December 2023. The chosen tool was the quality-of-life questionnaire for children: Continence Specific Pediatric Quality of Life Measurement Tool (PinQ), recommended by the ICCS,^1^ developed and validated by Bower et al.^9^ and Bower.^10^ The translation and cultural adaptation followed Beaton's model,^15^ consisting of six steps described as follows:

1. Initial translation: two translations of the original document in English were made into Portuguese.
2. Synthesis of translations: the versions were compared and synthesized into a single questionnaire.
3. Back-translation: after the synthesis of the questionnaire was completed, two independent translators who were native English speakers translated it into their source language, without access to the original questionnaire.
4. Committee of experts: a committee of experts was formed to discuss and produce the final version of the PinQ questionnaire. The committee of experts evaluated the versions of the questionnaire, analyzed the appropriateness of the responses and the translation of the questionnaire in terms of linguistic/idiomatic, conceptual, semantic, and cultural equivalence. In this way, the necessary items were modified, and the final version of the questionnaire was consolidated.
5. Prefinal version testing: to test this final version, we conducted a pilot study with 35 children (with their parents and/or guardians) that completed a standardized questionnaire, thus ensuring that the adapted version still maintains its semantic, linguistic, and cultural equivalence in an applied situation.
6. Submission of documentation to developers: the final phase of the adaptation process is the presentation of all reports and forms to the instrument developer who will verify whether the recommended steps were followed and verify that the content and objectives of the tools were maintained during the translation and validation process.
7. Validation: the final version of the questionnaire was administered to 20 children after the parents and/or guardians read and signed the Free and Informed Consent Form. The children completed the questionnaire alone, asking the researcher for help in cases of doubt.

To evaluate sample data, descriptive and inferential statistical methods were applied. Quantitative variables were presented by measures of central tendency and variation, and their normality was assessed using the Shapiro-Wilk test. To compare quantitative variables, Student's t-test for independent samples was applied. Pearson's correlation was applied to assess correspondence between quantitative variables. The alpha error was previously set at 5% for rejection of the null hypothesis, and statistical processing was performed using the BioEstat version 5.3 and SPSS version 27.

Patients underwent 15 individualized physiotherapy sessions. The first session was an evaluation, including a patient assessment with history-taking and the application of questionnaires:

1. The PinQ questionnaire aims to assess the quality of life of children and adolescents with urinary dysfunctions. The items assessed in this questionnaire are urinary symptoms, intestinal symptoms, social function, concerns, emotions, body image, and relationship with family. The questionnaire consisted of 20 questions. Scores range from 0 to 4, with "0" corresponding to "No" and "4" to "Always." The maximum score is 80 points. There is no set score, but the higher the score, the greater the impact on quality of life.^9,10^
2. The DVSS questionnaire aims to assess the frequency and severity of symptoms related to voiding dysfunction in children and adolescents, aiding in the diagnosis of bladder and bowel dysfunction. The questionnaire addresses several aspects of urination and bowel function, including: daytime and nighttime urinary frequency, urinary urgency, urinary incontinence, straining to urinate, interrupted or weak urinary flow, sensation of incomplete emptying, and constipation. In the score, each item has response options with predefined scores. The total score is obtained by adding the points of all responses. A higher score generally indicates greater severity of symptoms of voiding and bowel dysfunction. Specific cutoff points can be used to classify the severity of the dysfunction.^13^

The questionnaires mentioned above were applied by the evaluator and the child, without interference from the guardians. At the end of the evaluation, a urinary diary was provided to the guardian for two days, along with the following instructions for its completion: recording the exact time the child urinated, qualifying the volume urinated in a graduated container (in ml), recording the fluid intake, type and quantity (in ml) drunk, and the time, information on urinary losses (recording the time and quantity, whether small, moderate, or large).

The electrostimulation equipment used was the Dualpex 961. Children were positioned prone, and the sacral region (S2–S4) was cleaned with 70% alcohol for the placement of adhesive electrodes. The device was programmed with a frequency (F) of 10 Hz, pulse width of 700 μs, and intensity according to the sensory threshold,^16^ possible adverse effects of electrostimulation may include: skin irritation, pain or discomfort, involuntary muscle contractions,s and allergic reactions.

From the second to the 15th treatment session, sacral neurostimulation was performed using the Dualpex 961, programmed for 30 min; frequency (F): 10 Hz; pulse width (μs): 700 μs, and intensity (i): according to the patient's sensory threshold. In the second session, we established the patient's routine based on the voiding diary feedback, starting urotherapy with scheduled voids and fluid intake volumes according to the data filled out by the patient. In this same session, the patient learned the proper positioning for voiding and defecation. In the third session, we began with central stabilization associated with diaphragm and transverse abdominal muscle recruitment, and pelvic floor muscle activation, along with electrostimulation.

From the fourth to the eighth sessions, we continued with central stabilization associated with diaphragm and transverse abdominal muscle recruitment and pelvic floor muscle activation, combined with exercises such as sustained pelvic lifts for 5 s (three sets of 10 repetitions); bridge exercises with a disc (three sets of 10 repetitions); and unilateral lower limb elevation (three sets of 10 repetitions).

From the ninth to the 14th sessions, we maintained central stabilization associated with diaphragm and transverse abdominal muscle recruitment and pelvic floor muscle activation, combined with sustained pelvic lifts for 5 s (three sets of 10 repetitions); bridge exercises with a disc (three sets of 10 repetitions); unilateral lower limb elevation (four sets of 10 repetitions); one-legged balance training (four sets of 10 repetitions), with one foot in front of the other combined with playful activities using a ball (four sets of 10 repetitions); balance training on a disc with playful activities using a ball (four sets of 10 repetitions); and vestibular training.

## RESULTS

A total of 300 sessions were conducted with 20 patients, with each patient receiving 15 weekly sessions. The mean age of the individuals in this group was 8.95 years. Most participants were male (65%), and female participants totaled 35%. Sociodemographic and clinical data were presented in Table 1.

**Table 1:** Sociodemographic and clinical data.

Sociodemographic and clinical data	n (%)	Mean age (range) years
Female	7 (35)	9.42 (7–12)
Male	13 (65)	8.69 (6–11)
White	7 (35)	
Brown	8 (40)	
Black	5 (25)	
Monosymptomatic enuresis	1 (5)	
Nonmonosymptomatic enuresis	18 (90)	
Daytime incontinence	1 (5)	

Legend: N: number of individuals; %: number of individuals in percentage

**Figure 1:**
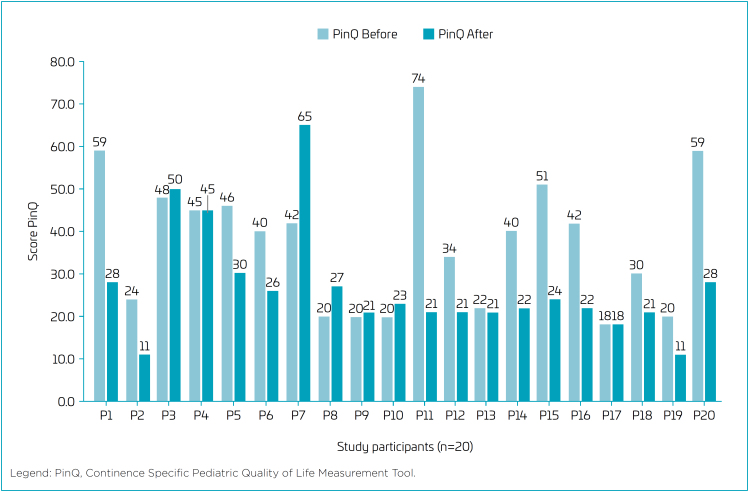
Continence Specific Pediatric Quality of Life Measurement Tool score, before and after assessments.

The PinQ Scores before and after treatment are demonstrated in Figure 1. The quality-of-life assessment using the PinQ questionnaire revealed a mean score of 37.7, with a standard deviation of 15.9 and a median of 40.0. Following the completion of 14 treatment sessions, children showed a mean score of 26.8, standard deviation of 12.9, and median of 22.5. The hypothesis test yielded a p-value of 0.0051*, which is statistically significant, indicating an improvement in the quality of life of the children.

**Figure 2:**
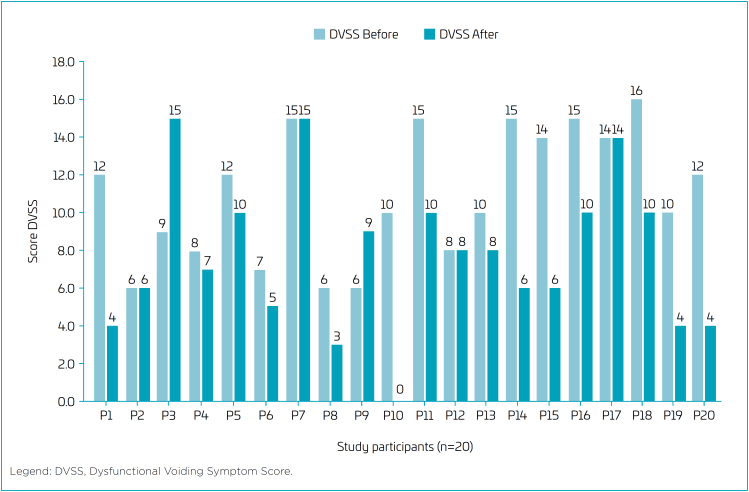
Dysfunctional Voiding Symptom Score, before and after assessments.

The DVSS scores are presented in Figure 2. The DVSS^12^ questionnaire showed that the 20 children evaluated before the intervention had a mean score of 11.0, standard deviation of 3.4, and median of 11.0, while after completing 14 treatment sessions, they presented a mean score of 7.7, standard deviation of 4.0, and median of 7.5. The hypothesis test yielded a p-value of 0.0055*, which is statistically significant, indicating an improvement in symptoms.

The correspondence between PinQ and DVSS was evaluated using Pearson's Linear Correlation, yielding a correlation coefficient of r=0.4190 with a nonsignificant p-value of 0.0656 before the intervention, and a correlation coefficient of r=0.4649 with a significant p-value of 0.0388 after treatment, confirming a positive relationship between PinQ and DVSS, as shown in Table 2.

**Table 2:** Continence Specific Pediatric Quality of Life Measurement Tool and Dysfunctional Voiding Symptom Score correlation.

	PinQ x DVSS Correlation	
	Before	After
Pearson coefficient	0.4190	0.4649
95%CI	-0.03 to 0.73	0.04 to 0.74
p-value	0.0656	0.0388
Test result	Not significant	Statistically significant

Legend: PinQ, Continence Specific Pediatric Quality of Life Measurement Tool; DVSS, Dysfunctional Voiding Symptom Score.

## DISCUSSION

Questionnaires are fundamental instruments for assessing the quality of life; however, it was noted that there was no specific tool for assessing the quality of life of patients with LUTD in Brazil. The PinQ questionnaire is a tool created and recommended by the ICCS^1^, and it is the first self-administered questionnaire addressing social and psychological aspects crucial for the quality of life of this specific population. The original questionnaire was created and validated in English and was translated and adapted to Brazilian culture following Beaton's proposed translation model.^15^ Currently, more rigorous methods for evaluating the translation and cultural adaptation process exist, such as the model proposed by Herdman et al.^17^ and adapted by Reichenheim & Moraes,^18^ which involves assessing conceptual equivalence and item semantics and operationality.

Reichenheim & Moraes,^18^ in his adapted version, proposed two steps: the first version of the questionnaire is applied, and after this application, a discussion with specialists takes place, consolidating a new translated and adapted version. Other authors who have conducted translation processes for questionnaires or evaluation tools originally created and validated in another language highlight the necessity of conducting transcultural adaptations, such as Rizzini et al.^19^ and Prado.^20^ These authors encountered challenges during the adaptation process and confirmed the relevance of assessing equivalence between the original instrument and its translated version.

During the translation and transcultural adaptation process of the PinQ-br questionnaire, several adaptations were made, including question 9: "sleeping away from home at a sleepover or holiday." Bachmann et al.^3^ encountered the same difficulty in translating this question into German. When adapting for German culture, the question was divided into two parts:

- Does my bladder problem prevent me from sleeping?
- Does my bladder problem prevent me from going on vacation?

Thus, the German version contains 21 questions instead of 20 as in the original version. In the translated version of PinQ-br for the Brazilian population, this cultural difficulty was not encountered, and it was translated as follows:

- Does my bladder problem prevent me from sleeping away on weekends?

This example underscores the need for the translation and cultural adaptation stages of questionnaires for the Brazilian population^21^ to facilitate patient understanding and increase the potential applicability of an instrument, despite the modifications made to the questionnaire being considered minor.

The PinQ brazilian questionnaire proved to be easy and straightforward to apply, demonstrating good reliability, internal consistency, and reproducibility, without requiring adaptations in the manner of completion or in the adverbs of time corresponding to the answers (never, almost never, sometimes, almost always, and always).^14^

The compatibility of the PinQ and DVSS^13^ brazilians questionnaires was evaluated using Pearson's linear correlation method, which before intervention showed a correspondence between the questionnaires with a correlation coefficient of r=0.4190 and p=0.0388, and after 14 weeks of treatment, the correspondence was r=0.4649 with p=0.0388, which is statistically significant, positively attesting to the relationship between PinQ and DVSS brazilian versions. When we tested the viability of each questionnaire, we observed that before the intervention, they had a mean score of 37.7, with a standard deviation of 15.9 and a median of 40.0. Upon re-evaluation after treatment, the scores changed to a mean of 26.8, standard deviation of 12.9, and median of 22.5. The hypothesis test yielded p=0.0051*, which is statistically significant, indicating an improvement in the quality of life of this population. A low coefficient may indicate that the items are not appropriate for what is intended to be evaluated — in this case, the quality of life of children with urinary dysfunction.^20^

This study demonstrates stability in the reproducibility of both questionnaires. Every assessment tool should yield the same results over time in two or more administrations to the same patient, provided their clinical condition has not changed.^15,21^ The structure of the original self-administered and proxy questionnaire, with the number of questions and scoring system, was considered perfectly suited to Brazilian culture and locale, without the need for adjustments as in the version translated by Bachmann et al.^3^ The PinQ brazilian demonstrated satisfactory internal consistency in both the self-administered version, as seen in the original instrument, and in the version translated by Bachmann et al.,^3^ with a Cronbach's alpha of 0.86, respectively.^22^

A possible limitation of this study refers to the actual influence of parents or guardians on the completion of the questionnaires; although it is a self-administered questionnaire answered by the children, there seemed to be some apprehension on the part of the child regarding their parents knowing their responses, making it difficult to assess how much their presence altered the indicated answers. A strong point of this research was its execution and duration, with a total of 300 sessions conducted, each lasting 60 min, totaling 18,000 h of clinical attendance.

We can conclude that both the PinQ and DVSS questionnaires are suitable tools that are easy to understand and complete. The PinQ questionnaire shows the real impact on the quality of life of these children and how it affects their daily activities and social life, allowing for a better understanding of the patient's condition. It should be noted that the DVSS questionnaire, while shorter and quicker to fill out, addresses more general issues focused on symptoms occurring over the past 30 days, not representing the complete context of quality of life concerning LUTD.

In conclusion, this study supports the effectiveness of a Portuguese-language tool that allows for the evaluation of the real impact of quality of life in children and adolescents with LUTD. Following up on this work, our plan is to study in the future how these problems really impact the lives of these children and adolescents, helping to develop even more research in this area.

## Data Availability

The database that originated the article is available with the corresponding author. CAAE: 52689921.4.0000.5505
